# Evaluation of in-service smartphone battery drainage profile for video calling feature in major apps

**DOI:** 10.1038/s41598-023-38859-3

**Published:** 2023-07-20

**Authors:** Hayder Ali, Hassan Abbas Khan, Michael Pecht

**Affiliations:** 1 Department of Electrical Engineering, SBA School of Science and Engineering, Lahore University of Management Sciences, Lahore, 54792 Pakistan; 2 Center for Advanced Life Cycle Engineering (CALCE), University of Maryland, College Park, MD 20742 USA

**Keywords:** Electrical and electronic engineering, Energy science and technology, Engineering

## Abstract

Video calling is one of the most energy-intensive features in apps requiring the simultaneous operation of the mobile camera, display screen, audio speaker, and internet services. This feature impacts a smartphone battery's runtime and lifetime. This paper is the first of its kind experimental study, which quantifies the operating profile (discharge current, temperature, and terminal voltage) of video call feature in multiple widely used social media apps, which include WhatsApp, Facebook Messenger, Zoom, Skype, WeChat, Google Hangouts, Imo and Viber. One smartphone each of Vivo and Motorola has been evaluated as the manufacturer-provided application programming interface (API) allowed real-time measurement of the operating profile. Results indicate that the video calling feature for Facebook Messenger and Imo is the most energy efficient. In contrast, Google Hangouts is up to 35% more energy-intensive for video calling than other apps. Measurements also show that Vivo's in-service battery temperature is lower than Motorola due to its efficient chipset. For instance, during active Google Hangouts operation for 1 h, Vivo temperature is limited to 46 °C, whereas Motorola temperature rises to 52 °C. Finally, the influence of app algorithms and codecs on energy efficiency is also discussed with regard to operating performance.

## Introduction

The requirements for video call applications (apps) on smartphones have increased for meetings and lectures in both educational and industrial sectors^1,2^. As a result, video calling and video chat apps have seen a significant rise in the market^3^. A record 62 million downloads of video calling apps were reported in March 2020^4^. Zoom led the way as its meeting attendees increased from 10 million in December 2019 to 300 million in April 2020^5^. With this client boost, the company's quarterly revenue in 2023 increased more than 1.07 billion dollars, which is 600% greater than the revenue in 2019^6^.

With increased video call operations, smartphone performance requirements have also increased. As the video call feature uses the camera, speaker, microphone, and WiFi (or mobile internet), the video calling app significantly reduces the runtime of the smartphone battery^7,8^. Further, the reduced runtime will likely result in larger cyclic requirements impacting the requirements for sizing batteries and determining battery warranties^9^. Additionally, energy-intensive apps can accelerate battery capacity fade by consuming discharge currents greater than 0.6C and causing elevated operating temperatures above 60 °C^10^. This can lead to degraded smartphone performance and even catastrophic failures^10,11^.

Many performance degradations, unexpected shutdown and even battery explosion cases have been reported in recent years. For instance, Apple admitted that it did slow down certain iPhones, but stated that it did so to extend the phones' life. Apple mentioned that when the lithium-ion batteries (LIBs) in the devices aged, they could not meet peak current needs. Consequently, an iPhone may shut down abruptly to safeguard its electrical components^12,13^. To settle the “Batterygate” case in 2020, Apple agreed to pay $113 million to customers affected by the underperforming iPhone models^13^. Similarly, the Italian Authority for Market and Competition fined Samsung $5.7 million in 2018^14^. In the most recent lawsuit (July 2021), the Spanish Consumer Protection Organization accused Apple of slowing down iPhone smartphones^15^.

Along with degraded performance, numerous customers have reported battery explosions in normal conditions. In 2021, a man sued Apple for second-degree leg burns caused by an exploding iPhone^16^. According to reports, various Samsung^17,18^ and Xiaomi^19^ models also exploded in the same year. Smartphone manufacturers typically recommend an operating temperature of no more than 45 °C^20^, whereas certain video calling and gaming applications can raise the smartphone temperature to 60 °C^7^. Due to energy-intensive features such as video calling and gaming, the chipset may become overloaded, resulting in increased current consumption and a subsequent rise in temperature above 50–60 °C^10^. This increase in temperature, combined with an increase in battery degradation, can lead to catastrophic failures, such as battery explosions^21,22^. Therefore, to evaluate the resource efficiency and operation of various features of an app, it is essential to obtain the application's discharge profile (current, voltage, and temperature) during its operation on in-service smartphones^23^.

Some studies have evaluated the in-service functionalities of smartphones. For instance, Wattenbach et al.^24^ compare the energy usage of Google Meet and Zoom using the software-based measurement tool Batterystats without hardware confirmations. In addition, the results provide the average energy consumption of two apps but do not provide information on the actual discharge current or temperature rise during operation. Trestian et al.^25^ evaluated Android video stream power consumption at a range of playback quality settings, video codecs in different scenarios. The results demonstrate that energy consumption is determined by signal quality and network load, followed by codec and playback quality. However, the results are obtained from the average energy consumption value and do not give information on the actual discharge profile of video calling apps. Anwar et al.^26^ also present a study that evaluates the influence of code structure on the energy consumption of Android apps. They demonstrated that by optimising the code's structure, energy consumption may be reduced by up to 10.8%. Kang et al.^7^ also presented a study that evaluated the thermal performance of smartphones during the operation of various apps/features such as Google Hangouts, Skype, video recording, Player Unknown's Battlegrounds (PUBG), and others. However, the study did not establish a correlation between the current drawn and the smartphone's battery life. Similarly, Zhang et al.^27^ conducted a thermal analysis for virtual and augmented reality apps without considering the runtime or battery health.

Existing research on in-service smartphone operation is limited, with many papers presenting analysis on controlled charge/discharge of smartphone batteries, which does not readily capture or emulate the field conditions with actual app usage for consumers. For instance, Kim et al.^10^ showed a thermal analysis of smartphones on fixed C-rates from 0.4C to 1C with a maximum temperature of 98.56 °C, much higher than the recommended temperature limit of 65 °C for LIBs. Nascimento et al.^28^ also presented controlled environment research in which they monitored external temperature changes in a smartphone battery during testing under different environmental conditions (dry, temperate, and cold) at constant charge and different discharge rates (1.32C, 2.67, and 5.77C). According to the cycling tests, when the battery is exposed to a cold environment, the battery performed the same charge/discharge cycles in 35% less time compared to dry and temperate environments. Further, Kwak et al.^29^ and Sun et al.^30^ compare and optimize the capacity degradation for three smartphone battery chemistries at different C-rates. While the aforementioned studies are useful, they do not emulate in-service operations.

While most papers conduct tests on smartphones and their batteries in a controlled setting or at a constant C-rate, limited in-service smartphone analysis is presented in the literature primarily focusing on thermal analysis. To the best of the authors' knowledge, no feature-specific assessment of video calling apps exists, even though video calling is one of the most energy and thermally inefficient features^7^. This paper, therefore, evaluates the performance of smartphones during in-service operation by directly measuring the current discharge profile, terminal voltage, and battery temperature profile using internal sensors. The suggested approach leverages the manufacturers’ built-in mobile sensor data using an application program interface (API). This work does not incorporate indirect estimation or parameter averaging, and our earlier work presents the developed API and algorithm^11^. This paper analyzes how eight major video-calling apps (WhatsApp, Facebook Messenger, Zoom, Skype, WeChat, Google Hangouts, Imo and Viber) consume energy and impact a smartphone's runtime performance. The discharge profile affects the battery's life directly through the C-rate effect or indirectly through the combined temperature and current spike effect^9,31^.

This paper presents the experimental data (discharge current, voltage and temperature) of eight major video-calling apps for two smartphones (Vivo and Motorola), as the two manufacturers only provide the requisite APIs for limited models for in-service operation. Our earlier research on seven smartphone manufacturers—including Samsung, Google, Huawei, Oppo, Vivo, Motorola, and Xiaomi—discovered that all other major manufacturers do not provide user access to this data^11^. While there are ways to probe smartphones for in-service data externally, these methods add uncertainties and measurement errors, reducing the overall accuracy^32,33^. Therefore, the presented scheme in this work is the most accurate measurement for discharge profiles using only the smartphone's inherent sensors for data acquisition. In addition, this paper addresses a significant research gap in the field of smartphone battery characterization, where the lack of user-accessible data and reliable measurement methods for battery discharge profiles has hindered progress in the development of battery degradation models for smartphones and has limited the ability to optimize battery performance under different lifecycle conditions. The data obtained from this study is highly reliable (comes from internal smartphone sensors without retrofitting or external probing) and can be used to develop accurate battery degradation models that can aid in the optimization of battery performance for different smartphone models and lifecycle conditions. Furthermore, the results presented in this paper are also connected to the energy efficiency of video and audio codecs of video calling apps. Thus, this study provides valuable insights for researchers and developers in this field, enabling them to improve the energy efficiency of video and audio codecs and enhance the overall performance of video calling apps.

The rest of this paper is arranged as follows. "Methodology" discusses the evaluation methodology. "Results and discussion" provides the experimental data and discharge profile analysis of two smartphones under test. It also quantifies the smartphone's energy requirements, current spikes, and temperature for the eight video calling in-service operations. "Conclusions" presents the conclusions.

## Methodology

The availability of in-service battery data, such as current, voltage, and temperature, depends on manufacturer-supplied software interfaces and the functionality of a smartphone’s built-in sensor hardware. As the sensors are in-built, in this work, we utilize an Android Package Kit (APK) using the manufacturer-provided API^11^ to monitor the battery's current, voltage, and temperature. The data is subsequently saved in the smartphone's internal memory.

Resolution of the parameters and sampling frequency of the API update period is critical for correctly gauging parameter fluctuations. Our research found that almost all major manufacturers do not provide information at high granularity to analyze the detailed discharge profile of apps. The Vivo (V9) and Motorola (Droid Turbo) have been chosen for this analysis as they allow the suitable resolution of discharge current and the quickest update time^11^. Table 1 provides a summary of the update time and resolution for electrical current, terminal voltage, and temperature values for both smartphones. The values presented are derived from the average observations made during the experiments.

**Table 1:** Summary of the update time and resolution pertaining to electrical current, terminal voltage, and temperature values.

	APK value recorded time (s)	Value update time (s)		Resolution		
		Current	Voltage/temp	Current	Voltage	Temp
Vivo	0.003	0.01	38	µA	mV	0.1 °C
Motorola droid turbo	0.0065	0.175	40			

This study employed smartphones in an excellent operational state to conduct targeted assessments exclusively for experimental purposes rather than general usage. The battery capacities of these smartphones were examined by subjecting them to three complete discharge cycles, from 100 to 0%, at a temperature of 25 °C. The outcomes revealed that both smartphone batteries exhibited an approximate discharge capacity of 98% compared to their initial nameplate capacities at the time of conducting the experiments. These findings imply that both smartphones met acceptable benchmarks, indicating that the batteries had not experienced significant capacity deterioration due to calendar or cyclic aging. Based on these results, smartphones are considered suitable for video call experiments, which require consistent battery performance over an extended period.

To evaluate the discharge profile, the Vivo (V9) and Motorola (Droid Turbo) phones were initially charged to their maximum battery capacity (100%) to ensure consistent terminal potential across all experiments. Following this, each video calling app was operated for 60 min and then charged back to full capacity. Consequently, the phone was discharged with the second test app, and so on. During this time, only the video call function of the particular app was in play with experimental conditions held constant to mitigate any heterogeneity between apps. During each experiment, there was an approximate 10–15 min rest period between charging and discharging. All apps were evaluated via WiFi connectivity, and the internal speaker sound volume and screen brightness were maintained at maximum levels with an ambient temperature of 26 ± 0.5 °C. It is noteworthy that renowned manufacturers of lithium-ion batteries, such as Samsung^34^ and Panasonic^35^, conduct their battery capacity evaluations at a temperature of 25 °C. Therefore, the experiments' environmental temperature of 26 ± 0.5 °C is appropriate.

In addition to the discharge profile, which includes current, terminal voltage, and temperature, the spread of current drainage provides valuable insights into the performance of video calling apps. To evaluate this spread, the probability density function (PDF) and the normal distribution function (NDF) were used. The PDF describes the probability distribution of all current levels generated by multiple video calling apps. The NDF is derived from the PDF and represents a normal distribution of the current levels. The peak value in the NDF represents the mean value of the current levels, while the standard deviation of the NDF represents the variability in the current levels. By recreating the current profile using these functions, it is possible to assess the smartphone's performance under various usage scenarios and identify potential areas for improvement. The PDF and NDF functions can be used to recreate the equivalent current profile of smartphones during video calling sessions. By applying the recreated current profile, it is possible to test the battery's degradation under varying environmental conditions, such as temperature, and identify any potential performance limitations. PDF and NDF can also be used to develop degradation models that predict the expected battery performance and estimate the battery's remaining lifespan. These models can be beneficial in optimizing battery life and improving overall smartphone performance.

In addition to app usage, battery drainage also depends on smartphone technologies such as screen type, resolution, and processor technology. For example, the Motorola Droid Turbo features a resolution of 565 PPI^36^, while the Vivo V9 has a resolution of 400 PPI^37^. Higher resolution corresponds to increased pixel density and consequently greater power consumption. Additionally, the Vivo V9 (14 nm technology^37^) utilizes a more energy-efficient processor than the Motorola Droid Turbo (28 nm technology^36^). Furthermore, other aspects, such as heat dissipation design, influence smartphone performance. Table 2 provides a technology comparison of both smartphones. The Vivo V9 exhibits superior performance and energy efficiency, albeit at a 35% higher cost^36,37^. However, it's inappropriate to compare the overall energy efficiency of the apps between both phones due to differences in hardware.

**Table 2:** Technology comparison of Vivo V9 and Motorola Droid Turbo.

Category	Feature	Vivo V9	Motorola droid turbo	Comments
Release year	–	2018^37^	2014^36^	–
Display	Type	IPS LCD	Super AMOLED	Owing to the presence of a backlight, IPS LCD consumes more energy than SUPER AMOLED. Furthermore, SUPER AMOLED is more expensive than IPS LCD. When it comes to picture and color contrast, people have ambivalent feelings^38,39^
	Size	99.1 cm^2^	74.5 cm^2^	A phone with a larger screen is generally preferred, but it can also consume more energy^40,41^
	Resolution	1080 × 2280 pixels (400 ppi)	1440 × 2560 pixels (565 ppi)	A smartphone with a lower resolution display can consume less power than one with a higher resolution display, although at the expense of a slight decrease in display quality^42,43^
Battery	Type	Lithium polymer	Lithium polymer	The manufacturers did not disclose the chemistry of the lithium-polymer batteries used in both smartphones
	Typical capacity	3260 mAh^37^	3900 mAh^36^	
Platform	Chipset	Qualcomm MSM8953-Pro Snapdragon 626 (14 nm)	Qualcomm APQ8084 Snapdragon 805 (28 nm)	The Vivo V9 platform outperforms the Motorola Droid Turbo platform in terms of performance and energy efficiency. Snapdragon 626, on the other hand, is nearly ten times more expensive than Snapdragon 805, owing to technical development^44^
	CPU	Octa-core 2.2 GHz Cortex-A53	Quad-core 2.7 GHz Krait 450	
	GPU	Adreno 506	Adreno 420	

## Results and discussion

The section examines the discharge characteristics of eight video calling applications as observed on the Vivo V9 and Motorola Droid Turbo smartphones. It also explores and categorizes the energy efficiency of these apps based on their algorithmic functionalities. Finally, it discusses each app according to its runtime performance on both devices.

### Usage profile of video calling apps

**Figure 1:**
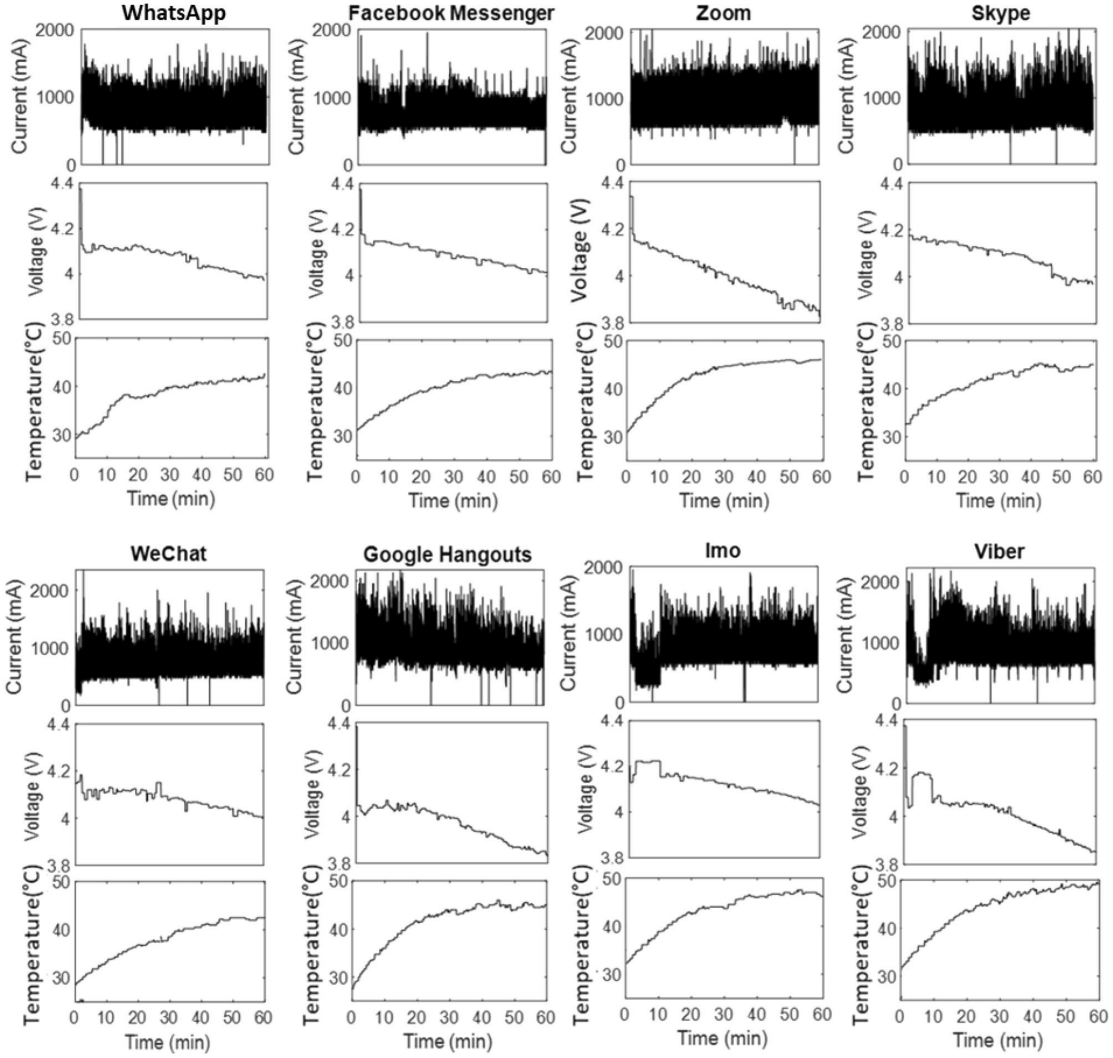
Discharge current, terminal voltage, and battery temperature profile for the Vivo V9 smartphone.

**Figure 2:**
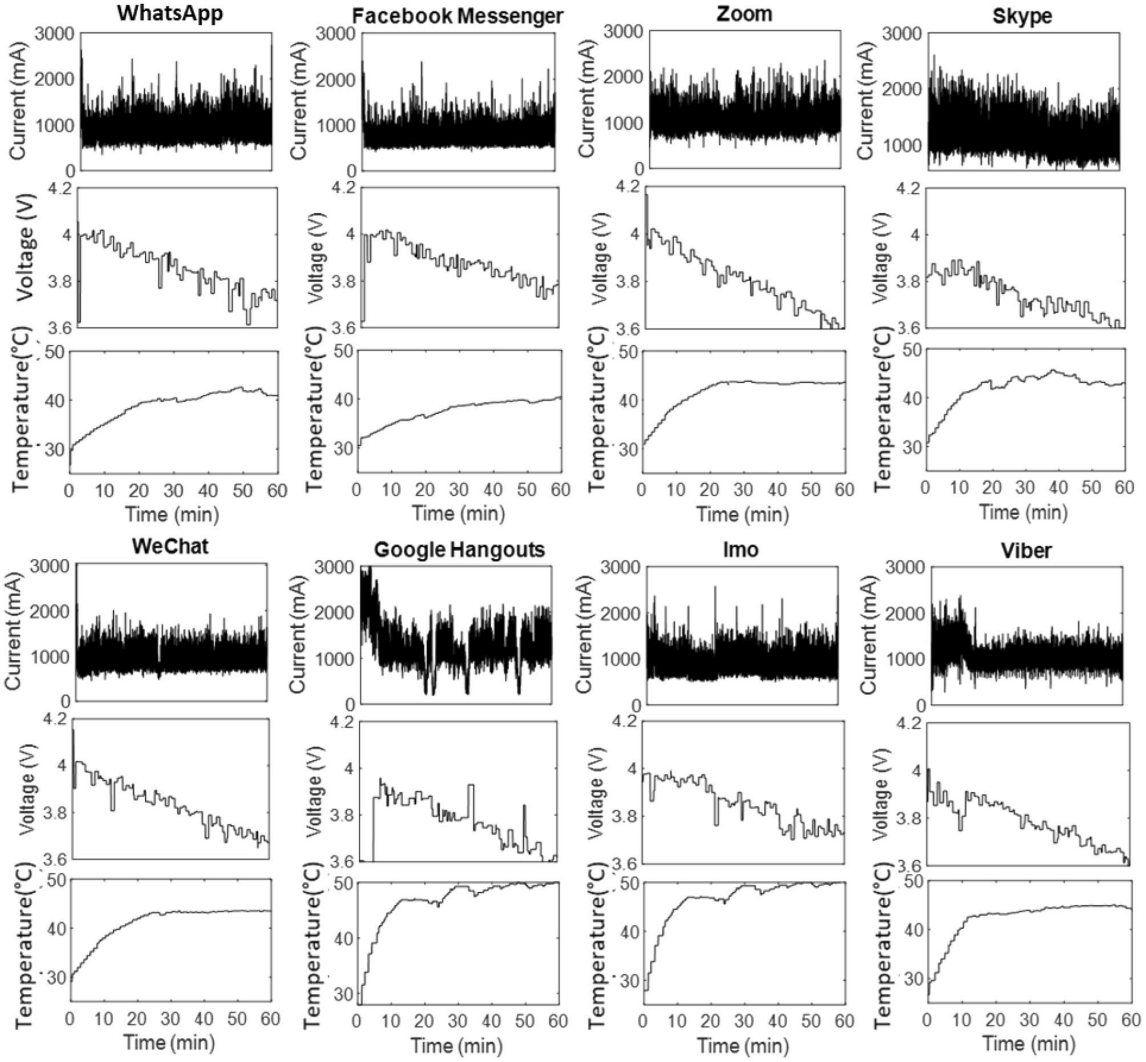
Discharge current, terminal voltage, and battery temperature profile for the Motorola Droid Turbo smartphone.

Figures 1 and 2 show the discharge profile for the video call feature during the in-service operation of the eight apps for both smartphones. The current drawn in peaks is in line with the feature operation of the apps, whereas the temperature and the terminal voltage change are more gradual. Each sensor has a different resolution and updating period as given by the manufacturer and outlined in Ref.^11^ where, for most manufacturers, the updating time of the voltage sensor is large (several seconds) relative to the current sensor (milliseconds). Each experiment had an approximate 15-min rest period between charging and discharging. At the start of each experiment, the battery surface temperature ranged from 30 to 35 °C, influenced by factors like chipset heat generation and could not be precisely controlled in a real-world scenario. Further, manufacturers such as Samsung demonstrated that the battery's nominal capacity remains consistent between temperatures of 25 °C and 35 °C^34,45^.

A higher starting current spike causes an initial voltage drop for some apps. For instance, in the cases of Google Hangout, the current is high at the start (> 2800 mA), then gradually drops to a lower value of around 1200 mA. As a result, the voltage profile has an initial voltage drop to 3.6 V followed by an increase to 3.9 V as the current decreases. Furthermore, the temperature rises steadily throughout the video call operation for both smartphones and becomes largely flat after 15–20 min of operation. For the Vivo V9, Google Hangouts, Imo, and Viber display the maximum temperature increase of 46 °C, 48 °C, and 49 °C, respectively. For Motorola, Google Hangouts accounts for the maximum rise in temperature of 52 °C, and Facebook Messenger accounts for the lowest temperature, remaining under 40 °C. Lower operational temperature is highly beneficial as it reduces the aging rate of the battery. The experimental readings of current, voltage, and temperature are provided in the supplementary file for both phones.

**Figure 3:**
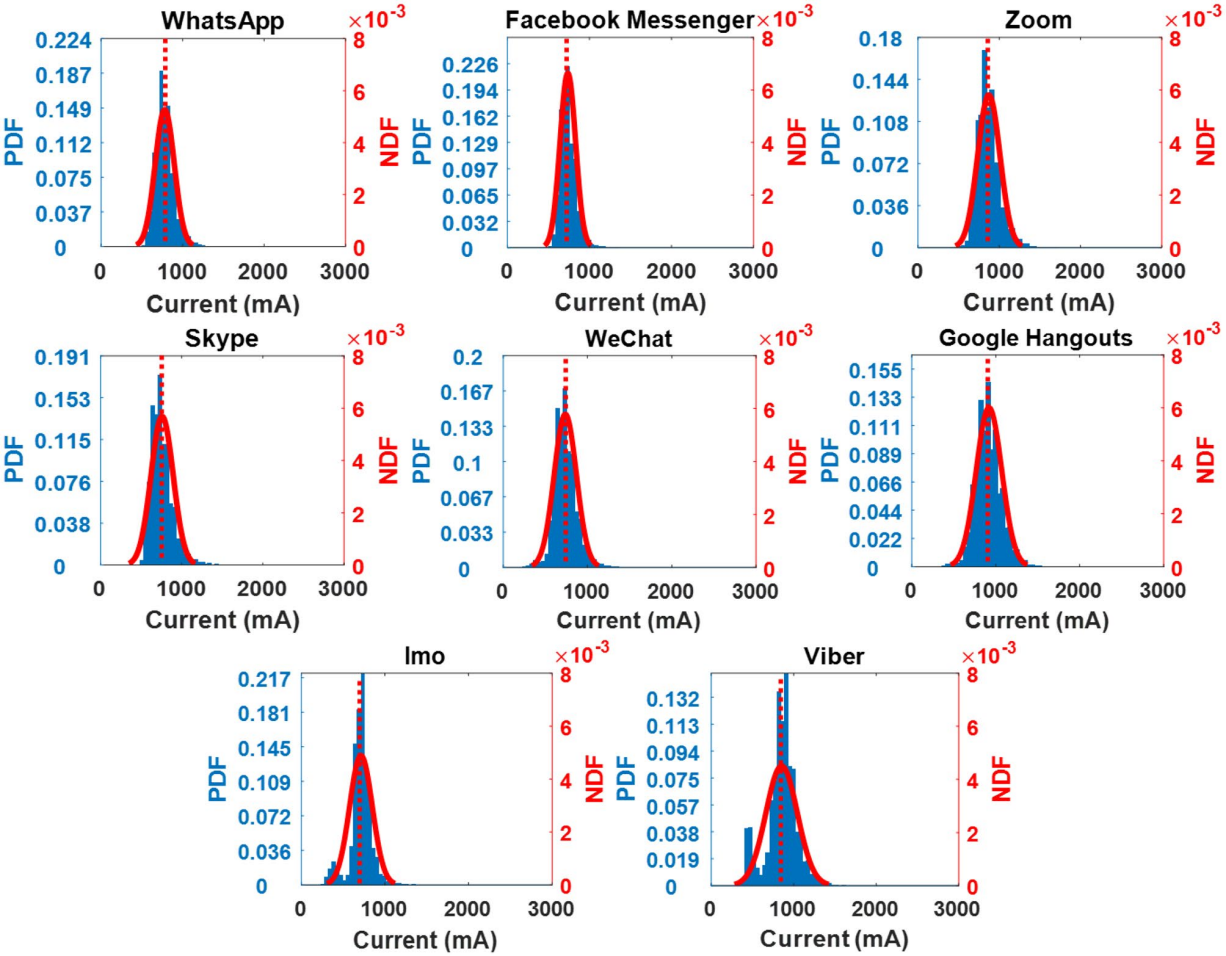
Probability density functions (PDFs) and normal distribution functions (NDFs) for all video calling apps for the Vivo V9.

**Figure 4:**
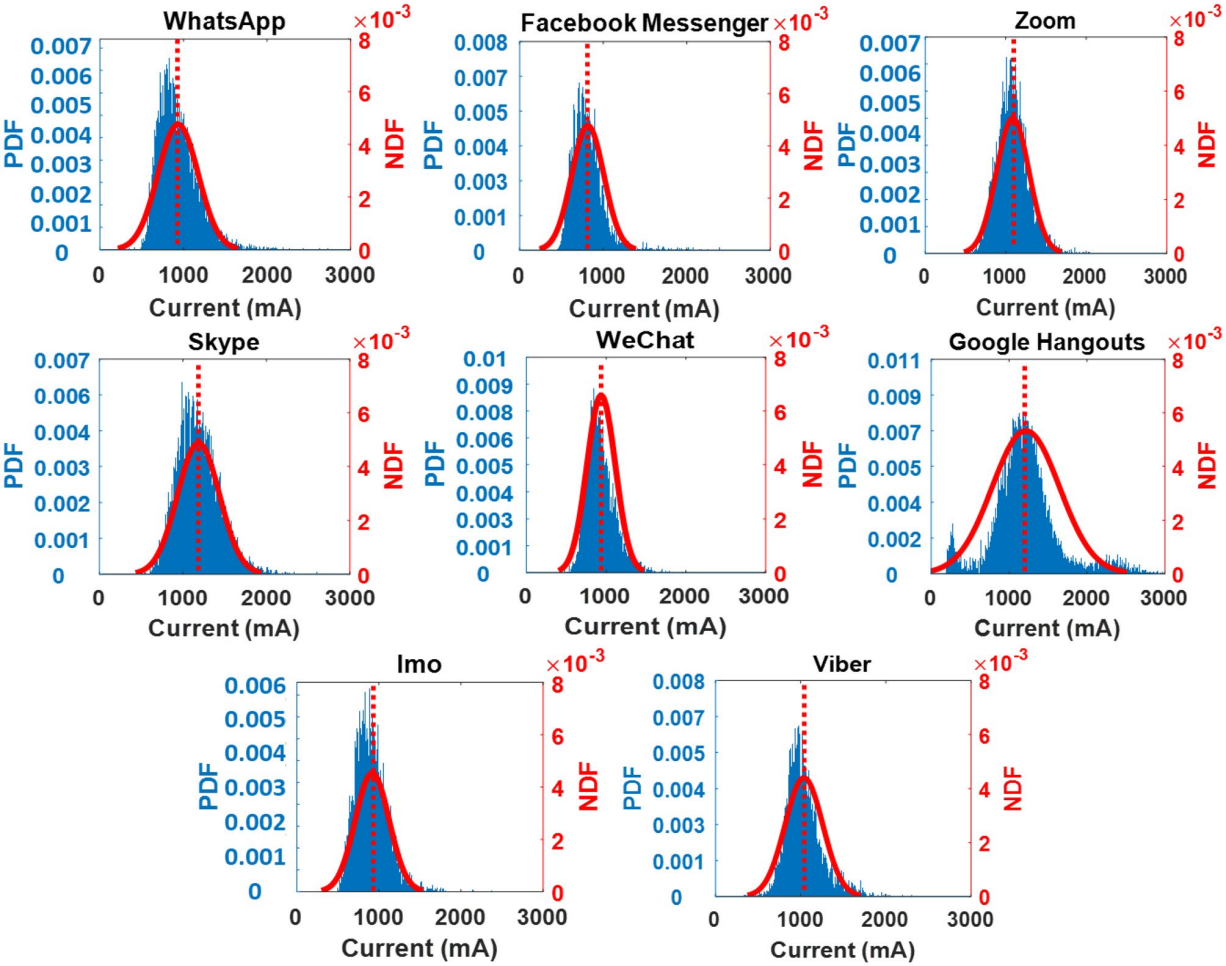
Probability density functions (PDFs) and normal distribution functions (NDFs) for all video calling apps for the Motorola Droid Turbo.

Relying only on the mean current discharge is insufficient for categorizing these apps. Current spike information is often essential as spikes cause an increase in temperature (joule loss) and accelerated battery deterioration^46^. To facilitate further analysis, the PDF and NDF are also presented in Figs. 3 and 4. It is important to highlight that the histogram bars of Motorola exhibit a narrower width in comparison to those of Vivo due to the utilization of more refined and precise measurements. Consequently, the discharge current readings are extensively distributed with thin bars across the current axis, as visually depicted in Fig. 4. In the case of the Vivo, Google Hangouts draws the highest current, around 919 mA (0.28C), with a standard deviation of 151 mA, as summarized in Table 3. On the other hand, Facebook Messenger, WeChat, and Imo draw the lowest mean current, approximately in the range of 730 mA. For the Vivo phone, Facebook Messenger, WeChat, and Imo use up to 30% less battery time than Google Hangouts and Zoom. For Motorola, Google Hangouts uses 1330 mA (0.34C) of mean current—considerably higher than the other video calling apps, as outlined in Table 4. Zoom and Skype require roughly 1100 mA (0.3C) mean current. In contrast, Facebook Messenger's mean current value is only 814 mA (0.2C), meaning that a video call on Facebook Messenger preserves 60% more battery time than Google Hangouts.

**Table 3:** Summary of current and temperature profile for the Vivo V9.

Features	Video call feature				
	Current profile				Temperature profile
	Mean current (mA)	Peak current (mA)	Standard deviation from mean current (mA)	Probability of current spike over 0.3C	Max temperature (°C)
Google hangouts	919 (0.28C)	2175 (0.67C)	151	34%	46
Zoom	857 (0.26C)	2044 (0.62C)	129	18%	46
Viber	853 (0.26C)	2219 (0.68C)	193	25%	49
WhatsApp	781 (0.24C)	1782 (0.55C)	122	6%	43
Skype	754 (0.23C)	2044 (0.62C)	135	5%	45
Facebook messenger	738 (0.22C)	1957 (0.60C)	98	1%	44
WeChat	727 (0.22C)	2350 (0.72C)	138	4%	43
Imo	704 (0.21C)	1957 (0.6C)	150	3%	48

**Table 4:** Summary of current and temperature profile for the Motorola Droid Turbo.

Features	Video call feature				
	Current profile				Temperature profile
	Mean current (mA)	Peak current (mA)	Standard deviation from mean current (mA)	Probability of current spike over 0.3C	Max temperature (°C)
Google hangouts	1330 (0.34C)	3075 (0.79C)	379	66%	52.1
Skype	1186 (0.3C)	2605 (0.67C)	251	53%	46
Zoom	1087 (0.3C)	2354 (0.6C)	202	34%	44
Viber	1037 (0.27C)	2382 (0.61)	220	27%	45
WeChat	936 (0.24C)	2152 (0.55C)	177	10%	44
WhatsApp	934 (0.24C)	2728 (0.7C)	240	17%	43
Imo	917 (0.23C)	2574 (0.66C)	205	11%	43
Facebook messenger	814 (0.2C)	2392 (0.6C)	192	3%	40

The mean current alone is not sufficient for the lifetime assessment of smartphone batteries. For instance, the average current requirement for Zoom is 857 mA (Table 3) compared to 853 mA for Viber. However, 25% of the current spikes in Viber usage in the phone is beyond 0.3C compared to Zoom, which only has 18% spikes beyond 0.3C. Therefore, Zoom operation is more conducive from a long-term degradation perspective than Viber. A higher mean current and high amplitude spike from the smartphone battery generates more heat and, thus degrading the battery health. Standard deviation (SD) values and mean currents are also described in Tables 3 and 4 to recreate current discharge profiles. This information serves as the baseline to evaluate app operation on smartphones for degradation assessment.

For optimum performance, smartphone manufacturers (such as Samsung^45^, LG^47^ and Sony^48^) recommend discharging the battery at up to 0.3C. In addition, numerous studies on various battery chemistries reveal a considerable capacity degradation above 0.3C^49,50^. Further, many studies also establish that C-rate operation over 0.3C results in battery temperature above 50 °C^51,52^, accelerating other reliability concerns such as solder joint failures^53,54^, thermal crack^55,56^ and electro-migration^56,57^. Therefore, a safe limit for LIB operation is set at 0.3C. So, to analyze the power usage for all eight apps, the probability of current spikes occurring above 0.3C is also shown in Tables 3 and 4 for Vivo and Motorola, respectively. The performance of apps differs significantly between Vivo and Motorola. For example, for Zoom on the Vivo smartphone, the mean current and the probability of current spikes above 0.3C is 857 mA (0.26C) and 18%, respectively, which is better than Zoom operation on the Motorola with a mean current of 1087 mA (0.3C) and the spike probability of 34%. High spike current drawn is related to multiple well-documented problems, such as deterioration to capacity and abrupt shutdowns^30,58^.

**Figure 5:**
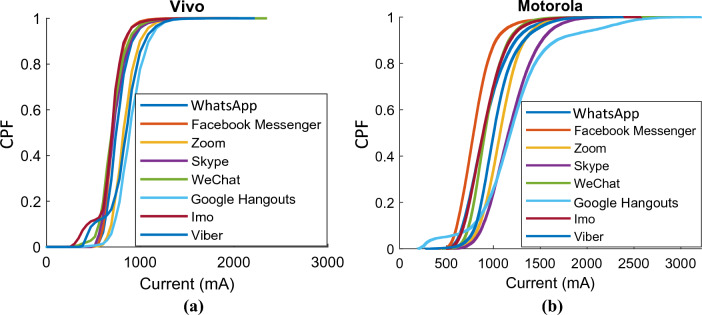
Cumulative probability function (CPF) for all video calling apps (**a**) Vivo V9 and (**b**) Motorola Droid Turbo.

For the Motorola device, WhatsApp’s video call efficiency is comparatively higher than Skype and WeChat, as shown in Table 4. The mean current for WhatsApp is 934 mA (0.24C), compared to 1186 mA (0.3C) for Skype and 936 mA (0.24C) for WeChat. Furthermore, the current spike probability for WhatsApp is lower at 17% compared to 53% for Skype. The cumulative probability function (CPF) for WhatsApp (and other apps) is plotted in Fig. 5 to compare the current discharge profile with the probability of spike occurrence. To calculate the CPF, the PDF is integrated over its domain. The CPF represents the probability that the random variable (in this case, spike amplitude) will be less than or equal to a specific value. The CPF is helpful in analyzing the behavior of a random variable over time and can help identify the likelihood of certain events occurring. In this case, the CPF also exhibits the variation in spike amplitude for all video calling apps. A stretch around the x-axis (current axis) indicates a greater probability of (unfavorable) high amplitude spikes. Consequently, Fig. 5 reveals that the efficiency of the Vivo V9 is superior to that of the Motorola Droid Turbo, due to a lower likelihood of unfavorable high amplitude spikes.

### Energy efficiency of the app's algorithm

The spread in Fig. 5 shows that the discharge profiles for all eight apps differ significantly across the two smartphones under test. This variation in discharge patterns between the two smartphones is attributed to the battery management system (BMS) and hardware (chipset and processor), as shown in Table 2. Additionally, the discharge profile may vary due to the app's algorithm, video/audio quality, and other active features during operation^59^. Various video calling apps employ several video and audio codecs, as indicated in Table 5, which can likely impact the total energy usage during operation. The mean current of both smartphones differs due to variations in technological parameters. To categorize the energy consumption of video and audio codecs, the average discharge C-rate of both smartphones is taken into consideration. In Table 5, the categorization is as follows: an average C-rate greater than 0.3C is labeled as high, the range of 0.26C to 0.3C is labeled as medium, and anything below 0.26C is labeled as low. For Google Hangout, the average discharge C-rate of both smartphones is 0.31C, which is the highest category. On the other hand, for IMO, it is 0.22C, which falls into the lowest category.

**Table 5:** Comparison of the energy efficiency of video and audio codecs used by various video calling apps.

Apps	Video codec	Audio codec	Energy consumption
Google hangouts	VP9^60^	G.711 and Opus^61^	High
Zoom	H.264^62,63^	G.711, G.722 and Opus^63^	Medium
Skype	RTVideo or H.264^64^	RTAudio, G.711, G.722 and Silk^64^	Medium
Viber	H.264^65^	G.729 and Opus^66^	Medium
WhatsApp	H.264^67^	Opus^68^	Low
Facebook messenger	H.264^69^	ISAC and Opus^70^	Low
WeChat	No data available	Silk^71^	Low
Imo	No data available	No data available	Low

Google Hangout employs the video codec VP9 and the audio codec G.711/Opus, which use 35% more energy, leading to better video and audio quality, as indicated in Table 5. Zoom and Skype employ the H.264 video codec, which is slightly inferior to VP9 video codec in terms of quality but may increase energy efficiency by up to 35%. Furthermore, it is also observed that apps using the Opus audio codec are more energy efficient than apps using the G.711/G.722 audio codec, as shown in Table 5. Most apps provide a combination of audio/video codecs and resolution and switch between them based on the strength of the available network connection, which also impacts energy consumption. The results retain their usefulness even if the app developer updates the apps and incorporates different audio and video codecs. In such situations, Table 5 can still assist in approximating the energy consumption, providing valuable insights for researchers and developers in this field. In addition to these, other factors such as echo (reflecting noises), may also affect the total energy usage throughout the operation.

### Runtime of video calling apps

**Figure 6:**
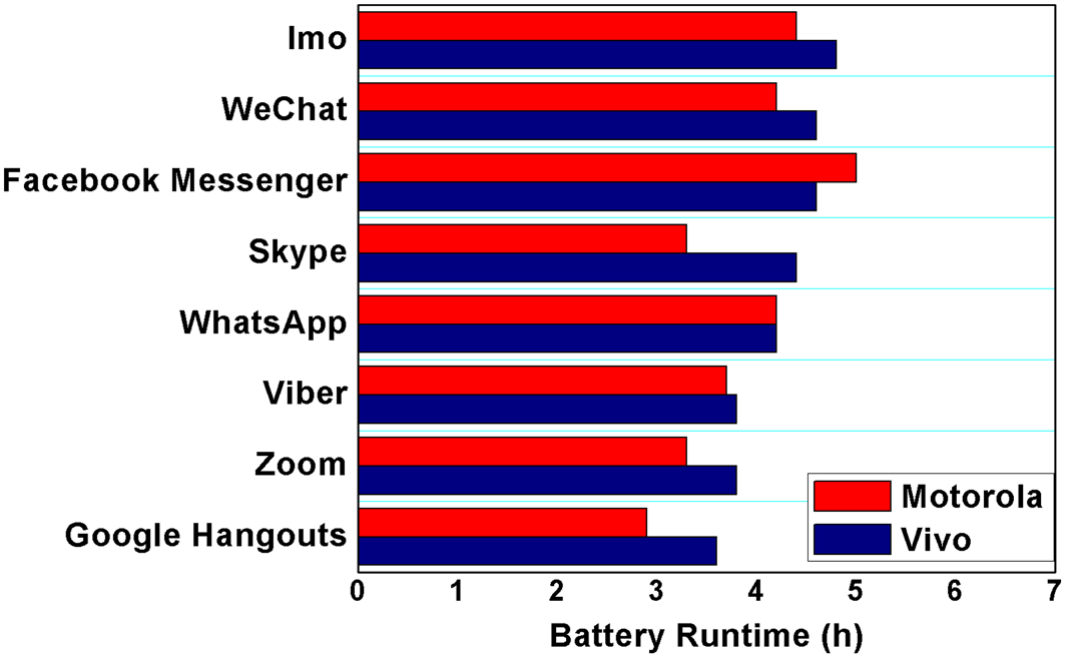
Battery runtime for all video calling apps with Vivo (3260 mAh) closely matching Motorola (3900 mAh) for certain apps, even with around 20% smaller battery capacity.

Energy-intensive apps reduce operation time in terms of smartphone battery capacity and runtime. For instance, for the Vivo V9 model with a 3260 mAh battery, runtime during video calls varied from 3.6 to 4.8 h (if no abrupt shutdown) for these eight different apps, as shown in Fig. 6. During video calls, Imo had the most extended battery runtime of 4.8 h, while Google Hangouts had the shortest battery runtime of 3.6 h. The runtime of the 3900 mAh battery ranged from 2.9 to 5 h for the Motorola Droid Turbo, as shown in Fig. 6. In this case, Facebook Messenger shows the most extended runtime of 5 h. In comparison, Google Hangouts is still the most power-extensive app, with a runtime of 2.9 h, 93% shorter than the manufacturer-suggested runtime of (up to) 48 h. Our empirical investigation revealed that the Vivo V9 outperformed the Motorola Droid Turbo by about 20%, attributed to its more energy-efficient hardware. However, in the context of Facebook Messenger, the Motorola Droid Turbo exhibited 5% longer run times compared to the Vivo V9. Our analysis indicates that the alteration in audio and video quality during the video call accounts for this trend.

## Conclusions

The paper evaluated the video calling functionality of two smartphones during in-service operation and, for the first time, quantified and reported the drainage profile of eight commonly used apps (WhatsApp, Facebook Messenger, Zoom, Skype, WeChat, Google Hangouts, Imo, and Viber) by comparing discharge profiles (i.e., current discharge, battery terminal voltage, and battery temperature). The two smartphone manufacturers (Vivo and Motorola) were selected for this study because they provide the requisite APIs for discharge profiles required for high granularity data shown in this work. The results of this study can be used to test battery degradation under different environmental conditions, identify performance limitations, and build a degradation model to predict battery lifespan and performance.

The discharge profile depends on the app requirements as well as the smartphone technology (e.g., screen type, resolution, and processor technology). For video calls on the Motorola smartphone, Google Hangouts was the most energy and temperature intensive, with a mean discharge current of 1330 mA (0.34C) and an end temperature of 52 °C for 1 h of operation. This was followed by Skype (1185 mA), Zoom (1087 mA), Viber (1037 mA), WeChat (936 mA), WhatsApp (934 mA), Imo (917 mA) and Facebook Messenger (814 mA). Along with mean current, information about current spikes is required to evaluate the impact of the discharge profile on battery life. For Google Hangouts and Skype, the probability of current spikes above 0.3C was more than 50%, significantly higher than all other apps. In the case of the Vivo smartphone, Google Hangouts was still the most energy-intensive app.

The results also indicate that the apps' audio and video codecs considerably impact energy usage during the operation. Regardless of the video or audio quality, apps using the VP9 video codec and G.711/G.722 audio use 35% more energy than apps utilizing the H.264 video codec and Opus audio codec. It also indicates that battery runtime numbers are significantly lower than the manufacturer-suggested phone runtime. For instance, for Motorola, the runtime of up to 48 h is misleading for many consumers; the actual runtime ranged from 2.9 to 5 h. Therefore, manufacturers should provide a time window (minimum to maximum) to give consumers more practical information on actual battery runtime.

Overall, results demonstrate that Google Hangouts is the most energy-intensive app for video calling for the two smartphones under test. On the other hand, the most energy-efficient apps for video calls are Facebook Messenger and Imo. The diversity in video and audio codecs across apps is the primary cause of the disparity in energy consumption.

The two smartphone manufacturers (Vivo and Motorola) were selected for this study because they provide the requisite APIs for discharge profiles required for high granularity data showed in this work. Other manufacturers, including Apple, Samsung, Google, Huawei, Oppo, Vivo, Motorola, and Xiaomi do not provide this data. We believe the manufacturers should be forthcoming in providing this data for third-party assessments leading to suitable suggestions for lifetime enhancements.

## Supplementary Information

Supplementary Information 1.

Supplementary Information 2.

Supplementary Information 3.

Supplementary Information 4.

Supplementary Information 5.

Supplementary Information 6.

Supplementary Information 7.

Supplementary Information 8.

Supplementary Information 9.

Supplementary Information 10.

Supplementary Information 11.

Supplementary Information 12.

Supplementary Information 13.

Supplementary Information 14.

Supplementary Information 15.

Supplementary Information 16.

## Data Availability

All data generated or analysed during this study are included in this published article and its supplementary information files.
